# The Future of Clinical Trials in Surgical Neuro-Oncology Is Bright

**DOI:** 10.3390/brainsci15121274

**Published:** 2025-11-27

**Authors:** Vratko Himic, Christian K. Ramsoomair, Sauson Soldozy, Ricardo J. Komotar, Michael E. Ivan, Ashish H. Shah

**Affiliations:** Department of Neurological Surgery, University of Miami Miller School of Medicine, Miami, FL 33136, USA

The field of surgical neuro-oncology continues to evolve at a rapid pace, driven by innovative advances from neurosurgeons, neuro-oncologists, and other experts across the clinical and basic sciences. This rapid progress reflects the field’s growing integration of molecular biology, precision imaging, and minimally invasive techniques, all of which are reshaping how we diagnose and treat central nervous system (CNS) tumors. The five papers featured in this Special Issue, “The Future of Clinical Trials in Surgical Neuro-Oncology”, reflect the breadth of strategies our colleagues are pursuing to advance treatment and improve outcomes for individuals diagnosed with CNS tumors. Together, these contributions highlight both the achievements to date and the critical need for continued innovation in trial design, multidisciplinary collaboration, and translational research to bring new therapies from the laboratory to the operating room and, ultimately, to patients.

Opening the discussion of the future of clinical trials in neuro-oncology, Shah and Heiss contributed a review on the future direction for improving therapies for glioblastoma (GBM) [1], a tumor for which effective therapies remain elusive and one whose standard of care and median overall survival have changed minimally over the past two decades [2]. Beginning with the history of GBM survival reports in the 20th century and followed by the role of the neurosurgeon in the care of patients with GBM, the authors then review the various treatment options and developments that have improved the arsenal of the neuro-oncological surgeon, including intraoperative stimulation mapping and awake craniotomy [3,4,5], intraoperative MRI [6,7], fluorescent labelling with 5-aminolevulinic acid (5-ALA) [8,9], and intraoperative Raman histology and laser-interstitial thermal therapy (LITT) [10], the latter of which enables a less invasive approach to treating GBM in certain cases. They finish with an update on novel therapies such as tumor-treating fields [11], immunotherapy, virotherapy, and the different delivery techniques that may enhance existing therapies, such as convection-enhanced delivery (CED) as well as methods to induce blood–brain barrier (BBB) disruption [1]. The authors concisely summarize the full breadth and depth of the GBM field and raise further questions that remain unanswered, all of which are ripe for future clinical trials.

Continuing with the theme of 5-ALA, Stummer and colleagues reported on a novel study design to evaluate the benefit of intra-operative fluorescence imaging [12]. Whilst previously approved for fluorescence-guided resection of gliomas, their article reports a multi-center prospective study design to better evaluate this surgical adjunct in meningioma surgery. Using 5-ALA (Gleolan™, NX Development Corps., Lexington, KY, USA), this phase 3 open-label single-arm trial (named NXDC-MEN-301) proposes administering oral 5-ALA three hours prior to surgery to all adult meningioma patients regardless of grade. The strength of this study proposal is the structured intra-operative schedule; they propose that the surgeon should first intraoperatively biopsy blue-fluorescing tissue and then move onto the white-light (conventional) surgery. Next, any “indeterminate” tissues (like the dural tail, near scar tissue, or at bony margins) are assessed using a presumed diagnosis and the extent to which they change their mind after the fluorescence light is used. This quizzing is then evaluated using post hoc histopathological confirmation. Finally, at the end of the case (once all white-light visible tumor has been resected), the blue light is once again used to identify remnant tissue. The authors extensively cover bias minimization (its sources and proposals for reduction) and the various limitations of the design. Study design articles that present ongoing and upcoming trials are at the center of neuro-oncological surgery clinical trial pipelines, providing an opportunity for peers and colleagues on the international stage to learn about, scrutinize, and collaborate on key developments in the field.

Beyond purely intra-axial tumors, skull base lesions remain a challenge for the neuro-oncology community. A key difficulty is finding the right balance between radical resection and maximum safe resection, particularly when the tumor is closely adhered to critical neurovascular structures. Kienzler et al. shared progress on adaptive hybrid surgery, whereby a planned subtotal resection is followed by stereotactic radiosurgery [13]. This can improve neurovascular structure preservation; for example, it can be used in vestibular schwannomas to preserve facial nerve function or target remote or residual tumor tissue too difficult to resect microsurgically [14]. They deployed an intra-operative tool that uses the Adaptive Hybrid Surgery Analysis method (AHSA, Brainlab^®^, Munich, Germany), building on their previously reported work [13]. Using tumor volumetric analysis of a cohort of five patients (composed of vestibular schwannoma, ependymoma, petroclival meningioma, and sphenoid wing meningioma), they were able to implement the following workflow: (1) the tumor was delineated as an “object”, after which other healthy tissue (brainstem, optic nerve, chiasm, the lens, etc.) were marked as so-called “Organs at Risk (OARs)” in a combination of automatic segmentation and surgeon-drawn residuals. The AHSA was employed preoperatively and then intra-operatively, where the radiosurgery and radiotherapy planning can be displayed alongside the tumor residuals in real time. At the end of this process, the AHSA software provides data that inform the team of whether the dose that was planned for the tumor and the OARs is too low, within a limit of acceptability based on the criteria and the maximum tolerated dose levels, or if it is sufficient. It provides these values for three potential treatment strategies: single fraction stereotactic radiosurgery (sf-SRS), hypofractionated stereotactic radiosurgery (hf-SRS), and conventional fractionated stereotactic radiotherapy (cf-SRT). In their case series, they show that safe hypofractionated radiation was achievable in all cases, and the patients experienced no complications in the 70-month average follow up period. Through illustrative cases, the authors show the power of using technology in the pre-, intra- and post-operative sphere to bring together the neurosurgical team, the radiation oncology team, and, ultimately, the patient’s anatomy and tumor burden to improve patient outcomes.

Continuing with the skull base theme, Jamshidi and colleagues reported on an exciting pilot study for the management of iatrogenic CSF leaks after endoscopic endonasal transsphenoidal resection (EEA) [15]. Both neurosurgeons and otorhinolaryngologists are wary of this relatively common complication whose treatment remains challenging; these iatrogenic leaks do not resolve with bedrest and often require CSF diversion and surgical revision [16]. Whilst lumbar drains (LDs) can be used, they are not without risk. A prior trial showed that acetazolamide (a carbonic anhydrase inhibitor) reduced the opening pressures by 10 cmH20, prompting Jamshidi et al. to attempt to use acetazolamide in this pilot trial. They combined high-volume lumbar puncture (LP) with 10 days of subsequent acetazolamide. In their four-patient study, all iatrogenic CSF leaks resolved by the second- and fourth-week follow-ups, with a 100% CSF leak cure rate and no complications. In this way, they were able to forego the invasive and complication-prone nature of LD insertion and replace it with LP and acetazolamide. Pilot studies such as these serve as the backbone for future larger prospective trials and lay the groundwork for addressing surgical complications in neuro-oncology.

Completing this Special Issue is a review from Yekula and colleagues on the role of single-cell RNA sequencing (scRNAseq) of CSF as an advanced form of liquid biopsy [17]. The promises of liquid biopsy to diagnose and monitor neurological disease have increased significantly, with progress made on both blood and CSF markers [18,19,20,21,22]. However, accompanying this promise are many logistical complications, including the relatively low CSF cell counts, strict timing and handling procedures, and how different isolation strategies (FACS, microfluidics, etc.) can dramatically skew what cell populations are analyzed [23,24]. They bring together the literature to summarize the benefits of this exciting technology once the right cells at the right quality are introduced into a sequencing pipeline, showing that scRNAseq of CSF can be deployed in neuro-oncology to understand the following: tumor heterogeneity in leptomeningeal metastasis [22], metabolic adaptations of circular tumor cells (CTCs) in the CSF compartment [25], immune remodeling during checkpoint inhibition [26], and the emergence of rare or disease-specific immune subsets in conditions such as multiple sclerosis, Alzheimer’s disease, COVID-related neuroinflammation, and HIV/AIDS. The overall message is that CSF scRNA-seq is already revealing biological features that bulk CSF tests simply cannot. However, the field is still in its infancy; different groups use different workflows, sample sizes are small, and standardization is urgently needed in order to increase the clinical reliability of this method. The rapid technological advancements in this space provide hope for the improved diagnosis and monitoring of a large spectrum of neurological disorders; scRNAseq of CTCs in CSF has a promising role in future clinical trials, as a form of treatment group sub-setting, a form of monitoring, or indeed a form of treatment personalization, paving the way for personalized neuro-oncological therapies.

To conclude, the future of clinical trials in surgical neuro-oncology is promising. Although not all neuro-oncology trials directly involve neurosurgical intervention, many would benefit from the integration of neurosurgical expertise, particularly studies focused on intraoperative interventions, biomarker development, and novel therapeutic delivery methods. The field has positioned itself at the forefront of innovation in imaging, molecular characterization, and targeted treatment strategies, advances made possible through sustained excellence in translational research. Nevertheless, significant challenges remain, particularly in improving outcomes for patients facing devastating diagnoses such as GBM. Continued progress will depend on strengthening the infrastructure that supports surgical neuro-oncology clinical trials. Leveraging established programs such as the Neurosurgeon Research Career Development Program (NRCDP K12), the Neurosurgery Research & Education Foundation (NREF), and the NIH Neurosurgeon–Scientist Training (NST) pipeline can accelerate this effort. Dedicated initiatives such as NREF continue to play a pivotal role in enabling pilot projects that evolve into larger externally funded studies [27]. Notably, every dollar invested by NREF in residents and early-career neurosurgeons has yielded approximately thirty-six dollars in subsequent NIH funding, underscoring the remarkable return on investment that targeted research support can achieve [28]. Advancing the next generation of surgical neuro-oncology trials will require vision, collaboration, and sustained support; but the foundation for meaningful progress is already firmly in place.
